# Draft Genome Sequences of 18 Strains of *Pseudomonas* Isolated from Kiwifruit Plants in New Zealand and Overseas

**DOI:** 10.1128/genomeA.00061-16

**Published:** 2016-04-07

**Authors:** Sandra B. Visnovsky, Mark Fiers, Ashley Lu, Preetinanda Panda, Robert Taylor, Andrew R. Pitman

**Affiliations:** a The New Zealand Institute for Plant & Food Research Ltd., Christchurch, New Zealand; b VIB Center for the Biology of Disease, Leuven, Belgium; c Plant Health and Environment Laboratory, Ministry for Primary Industries, Auckland, New Zealand; d Bio-Protection Research Centre, Lincoln University, Canterbury, New Zealand

## Abstract

In this paper, we present the draft sequences of 18 genetically diverse *Pseudomonas* strains isolated from kiwifruit plants in New Zealand and overseas, including a number that are currently not fully characterized. These sequences will aid in the diagnosis of *Pseudomonas* on kiwifruit for future pest management and border security decision-making.

## GENOME ANNOUNCEMENT

A recently emerged, highly virulent haplotype of *Pseudomonas syringae* pv. actinidiae biovar 3 causes severe stem cankers on kiwifruit (*Actinidia* spp.) associated with die-back of canes (1). Less virulent or nonpathogenic pseudomonads also exist on this host (S. B. Visnovsky, unpublished data), yet an understanding of *Pseudomonas* diversity and how it may impact disease outbreaks on kiwifruit remains unknown. We announce the draft genome sequences of 18 *Pseudomonas* strains isolated from kiwifruit plants, including isolates of *P. syringae*, *P. viridiflava, P. marginalis*, *P. fluorescens*, *P. putida*, and pseudomonads that are currently unassigned to a species (*Pseudomonas* sp.). Strains and their origins are listed in Table 1. Among the strains assigned to a species, we present the genomes of 15 strains obtained from the International Collection of Microorganisms from Plants (ICMP), two strains from the Culture Collection of Plant Pathogenic Bacteria in Wageningen, the Netherlands (PD), and a strain held at the New Zealand Institute for Plant & Food Research (NZIPFR) culture collection. The genomic data raise questions about the original taxonomic classification of several accessions. For example, ICMP 11289 and ICMP 13104, previously assigned to *P. marginalis* and *P. viridiflava*, are likely to be *P. viridiflava* and *P. cannabina*, respectively (S. B. Visnovsky and A. R. Pitman, unpublished data). Thus, the genome sequences of these 18 kiwifruit isolates will provide an opportunity for their reclassification and enable a better understanding of the genetic diversity of pseudomonads on kiwifruit in New Zealand and overseas. Greater understanding of their genetic diversity is important for future pest management, risk assessment of exotic organisms, and decision-making at the New Zealand border.

**TABLE 1:** Statistics for the 18 draft *Pseudomonas* genome sequences

Bacterial strain	Strain designation^a^	Accession no.	Host	Organ affected	No. of CDSs^c^	No. of scaffolds	*N*_50_ (bp)	Longest scaffold length (bp)
*P. syringae* pv. actinidiae biovar 4	ICMP 19497	LKBQ00000000	*A. chinensis*, Te Puke, New Zealand	Leaf	5,457	188	275,075	68,548
*P. syringae*	ICMP 11292	LKGU00000000	*A. deliciosa*, New Zealand, 1991	Bud	5,187	70	181,691	693,110
*P. syringae*	ICMP 11168	LKGV00000000	*A. deliciosa*, Katikati, New Zealand, 1991	Anther	5,078	97	135,412	393,655
*Pseudomonas* sp.	ICMP 10191	LKGW00000000	*Actinidia*, Changsha, China, 1981	Flower	5,345	189	182,604	360,761
*P. syringae*	ICMP 11293	LKEP00000000	*A. deliciosa*, Kumeu, New Zealand, 1991	Bud	5,454	60	253,227	637,394
*P. syringae*	ICMP 13102	LKEO00000000	*A. deliciosa*, Livron sur Drome, France, 1985	ND^b^	5,029	158	86,475	369,106
*P. syringae* pv. syringae	PD 2774	LKEL00000000	*A*. *chinensis*, USA	ND	5,404	180	75,729	311,946
*P. syringae*	ICMP 19498	LKCH00000000	*A*. *chinensis*, Te Puke, New Zealand, 2010	Leaf	5,872	109	118,302	366,337
*Pseudomonas* sp.	ICMP 3272	LKEK00000000	*A*. *deliciosa*, Riverhead, New Zealand, 1971	Bud	5,120	129	139,407	311,397
*P. syringae*	ICMP 19499	LKCI00000000	*A*. *chinensis*, Te Puke, New Zealand, 2011	Leaf	5,447	77	180,439	445,246
*P. syringae* pv. syringae	PD 2766	LKEM00000000	*A. chinensis*, USA	ND	4,728	105	133,143	482,177
*P. viridiflava*	ICMP 13104	LKEJ00000000	*A. deliciosa*, Begrolles en Mauges, France, 1985	ND	4,553	204	61,545	209,064
*P. marginalis*	ICMP 11289	LKGX00000000	*A. deliciosa*, Kumeu, New Zealand, 1991	Bud	5,369	134	111,563	257,942
*Pseudomonas* sp.	ICMP 19500	LKBK00000000	*A. chinensis*, Kerikeri, New Zealand, 2011	Leaf	5,778	144	118,097	274,028
*P. fluorescens*	ICMP 3636	LKEI00000000	*A. deliciosa*, New Zealand	Bud and blossom	5,852	101	144,381	377,916
*P. fluorescens*	ICMP 11288	LKEF00000000	*A. deliciosa*, Kumeu, New Zealand, 1991	Bud and flower	5,894	106	126,608	386,700
*P. marginalis*	ICMP 9505	LKGY00000000	*A. deliciosa*, Te Puke, New Zealand, 1987	Fruit	5,189	144	90,711	392,880
*P. putida*	ABAC8 (NZIPFR)	LKGZ00000000	*Actinidia*, New Zealand	ND	4,892	66	640,996	931,804

^a^ ICMP, isolates obtained from the International Collection of Microorganisms from Plants (http://www.landcareresearch.co.nz/resources/collections/icmp); PD, Culture Collection of Plant Pathogenic Bacteria, Plant Protection Service, Wageningen, the Netherlands; NZIPFR, isolates held in the New Zealand Institute for Plant & Food Research Ltd. culture collection.

^b^ ND, not determined.

^c^ CDSs, coding sequences.

Pure cultures of *Pseudomonas* strains were incubated overnight in Luria broth (LB) medium at 28°C. Genomic DNA was then isolated from each culture using a DNeasy Blood and Tissue kit (Qiagen) and each DNA sample was used to generate Illumina HiSeq 2000 100-bp paired-end reads (Centre for the Analysis of Genome Evolution and Function, University of Toronto, Canada). The quality of the sequence reads was checked using FastQC (Baraham Bioinformatics, United Kingdom) and low-quality reads (score of < Q30) were trimmed using Fastq-Mcf (https://code.google.com/archive/p/ea-utils/). A *de novo* assembly was then performed with the edited sequence reads using SOAPdenovo v4.1 (2) to generate draft genome sequences for the 18 strains of *Pseudomonas* with coverage of ~80×. Coding sequences for each isolate were annotated using the PGAAP annotation pipeline (http://www.ncbi.nlm.nih.gov/genome/annotation_prok/). A detailed phylogenetic and taxonomic study of these and previously published draft *Pseudomonas* sequences published in GenBank will follow.

### Nucleotide sequence accession numbers.

This whole-genome shotgun project has been deposited at DDBJ/EMBL/GenBank under the accession numbers listed in Table 1. The version described in this paper the first version.
